# *GBA*-Associated Synucleinopathies: Prime Candidates for Alpha-Synuclein Targeting Compounds

**DOI:** 10.3389/fcell.2020.562522

**Published:** 2020-09-25

**Authors:** Kathrin Brockmann

**Affiliations:** ^1^Center of Neurology, Department of Neurodegeneration and Hertie-Institute for Clinical Brain Research, University of Tübingen, Tübingen, Germany; ^2^German Center for Neurodegenerative Disease (DZNE), Bonn, Germany

**Keywords:** GBA, alpha-synuclein, dementia, GCase, Parkinson

## Abstract

With disease-modifying compounds targeting alpha-synuclein available in clinical trials, patient stratification according to alpha-synuclein-specific enrichment strategies is a much-needed prerequisite. Such a scenario will be exemplified for *GBA*, one major genetic risk factor that is specifically associated with the alpha-synucleinopathies: Parkinson’s disease and dementia with Lewy bodies.

## Introduction

Due to genetic studies in rare Mendelian cases of Parkinson’s disease (PD) 25 years ago, it became clear that the alpha-synuclein protein is the major component of Lewy bodies and Lewy neurites (Spillantini et al., 1997). It therefore plays a pivotal role in the pathogenesis of alpha-synucleinopathies such as PD and dementia with Lewy bodies (DLB). Subsequently, molecular pathways associated with alpha-synuclein clearance, aggregation, and propagation have been detected. Next to defects in vesicular trafficking, mitochondrial and importantly lysosomal dysfunction represent the most relevant pathways (Jankovic and Tan, 2020). Studying these likely early and initiating events provides “entry points” to develop novel therapeutic targets on an individualized basis.

Notably, histopathology in genetically associated forms of PD can differ. PD patients with *LRRK2* mutations show the typical Lewy-body pathology with alpha-synuclein aggregation but also tau aggregation or even nigral degeneration without distinctive histopathology. Nigral degeneration without Lewy body formation is also often seen in PD associated with biallelic mutations in the genes *parkin* or *PINK1* (Schneider and Alcalay, 2017; Henderson et al., 2019). With disease-modifying treatment options targeting alpha-synuclein under way, patient stratification according to alpha-synuclein-specific enrichment strategies as well as knowledge of the disease course and trajectories to disease-related milestones is a much-needed prerequisite to introduce patients to specific therapies. This will be exemplified for the gene *glucocerebrosidase (GBA)*, a major genetic risk factor for PD that is specifically associated with alpha-synuclein pathology.

### *GBA* Mutations Are a Major Genetic Risk Factor for PD

Biallelic mutations in the gene *GBA* cause Gaucher’s disease (GD), the most common lysosomal storage disorder with tissue accumulation of glucosylceramides due to deficiency of the lysosomal enzyme glucocerebrosidase (GCase). Interestingly, about 25% of GD patients report a first- or second-degree relative to present with typical Parkinsonism (Goker-Alpan et al., 2004; Halperin et al., 2006). This important clinical observation was the hint that heterozygous mutations in the *GBA* gene might be associated with PD. Subsequently, a large multi-center study across four continents analyzed 5691 PD patients of different ethnic origin compared to 4898 controls and confirmed that with an overall odds ratio (OR) of 5.43, heterozygous mutations in the *GBA* gene represent a major genetic risk factor for PD (Sidransky et al., 2009). This has now been confirmed across different ethno-racial populations with Caucasian, Asian (Japanese, Chinese, Taiwanese), Hispanic, and African ancestry (Neumann et al., 2009; Lesage et al., 2011; Chen et al., 2014; den Heijer et al., 2020; Mahungu et al., 2020).

Interestingly, some variants that have been reported as non-relevant for Gaucher disease (GD) have been proven to increase the risk for PD, e.g., p.E326K and p.T369M (Zhang et al., 2018; Iwaki et al., 2019). Consequently, *GBA*-subgroup classification for PD patients is often based on variant severity according to established genotype risks reported for PD.

Moreover, sequencing the *GBA* gene is very challenging due to the pseudogene. Further, one of the most common severe variants, p.L444P, is not covered well by standard genome-wide arrays such as Neurochip or NeuroXChip. Therefore, interpretation across different studies has to be done carefully, and the most comprehensive analyses are those done by whole gene Sanger sequencing.

### *GBA* Mutations and Parkinson Manifestation

#### *GBA*-Associated PD Presents With Non-motor Characteristics

Detailed investigation of the phenotypical spectrum of motor and non-motor symptoms is of utmost importance in order to design studies for disease-modifying therapies. PD patients with *GBA* mutations (PD_*GBA*_) show a younger age at onset with a median onset in the early fifties (Sidransky et al., 2009; Blauwendraat et al., 2019). Of note, this effect is not only attributable to *GBA* variants in general but is further driven by *GBA* mutation severity and mutation burden with most severe mutations as well as homozygous and compound heterozygous variants predisposing to the youngest age at onset (Thaler et al., 2017; Malek et al., 2018).

This is of importance as, in general, a younger age and age at onset are typically associated with a more benign disease course, especially in terms of cognitive decline (Forsaa et al., 2010). Keeping this in mind, other important clinical aspects have come to attention in PD_*GBA*_. Compared to sporadic PD patients without *GBA* mutation (PD_*GBA_wild type*_), PD_*GBA*_ present with a higher prevalence of cognitive impairment and more frequently suffer from additional non-motor symptoms including neuropsychiatric disturbances (depression, anxiety, hallucination), autonomic dysfunction, and sleep disturbances such as REM-sleep-behavior disorder (RBD) (Brockmann et al., 2011; Barrett et al., 2014). These findings have been replicated consistently over a the following years in other PD cohorts worldwide, the latest large clinical genome-wide association study in 4093 PD patients (Iwaki et al., 2019). Interestingly, variants that are classified as severe mutations (GBA_*severe*_) have been associated with a more aggressive clinical phenotype suggesting a relevant effect depending on *GBA* mutation severity (Cilia et al., 2016; Thaler et al., 2018; Petrucci et al., 2020).

Taken together, these clinical findings are of importance for the following reasons:

(1) In addition to demographics (age, age at onset, gender) and co-morbidities, they might offer explanations for the variability of the clinical phenotype in PD.
(2) They might provide defined temporal windows of phenotypical milestones to be addressed in disease-modifying trials beyond pure motor impairment.

#### *GBA*-Associated Parkinson’s Disease: More Rapid Progression and Shorter Survival in Prospective Longitudinal Studies

When it comes to clinical trials aiming at disease modification and not just pure symptomatic improvement, the rate of progression is crucial in order to estimate effect sizes and plan study designs (duration, sample sizes, etc.). Following up on these aspects, longitudinally investigated cohorts of PD_*GBA*_ revealed that this patient group, although younger in age and age at onset, present with an accelerated disease progression in terms of motor impairment, disease staging and cognitive decline. Moreover, survival rates are shorter when compared to PD_*GBA_wild type*_ (Brockmann et al., 2015b; Stoker et al., 2020).

#### Shorter and More Prominent Prodromal Phase in *GBA*-Associated PD

The typical motor manifestation of PD is preceded by a prodromal phase that is characterized by a variety of non-motor and early motor signs (Berg et al., 2015). Non-motor symptoms include amongst others hyposmia, autonomic dysfunction, and neuropsychiatric symptoms, whereas reduced arm swing and bradykinesia indicate early motor signs. However, type, prevalence, time of occurrence, and rate of progression of these prodromal symptoms vary between patients. Given the findings from the manifest disease phase in PD_*GBA*_ with pronounced non-motor symptoms and a more rapid disease progression, we retrospectively focused on patient’s perception regarding their individual prodromal phase before PD diagnosis. Comparing PD_*GBA*_ and PD_*GBA_wild type*_, we could show that: (1) prevalence and time of occurrence of prodromal symptoms seem more pronounced in PD_*GBA*_. They reported a shorter prodromal phase with almost parallel beginning of non-motor and early motor signs before PD diagnosis. Contrarily, PD_*GBA_wild type*_ showed a long prodromal interval starting with non-motor symptoms long before early motor signs manifested. (2) Patients carrying severe *GBA* mutations reported the highest total amount of prodromal signs. These findings suggest that clinical trajectories known from the manifest disease might be present already in the prodromal phase (Zimmermann et al., 2018). Indeed, prospective studies found that asymptomatic *GBA* mutation carriers present with parallel deterioration of non-motor and motor sign when compared to healthy controls without *GBA* mutation (Beavan et al., 2015; Avenali et al., 2019). There is only one prospective longitudinal study available in patients with RBD that monitored the prodromal phase until phenoconversion to manifest PD. While there were no differences in the severity of prodromal motor and non-motor markers, *GBA* mutation status was associated with accelerated phenoconversion to PD and/or dementia (Honeycutt et al., 2019).

### *GBA* Mutations and Dementia With Lewy Bodies

The important finding that PD_*GBA*_ more frequently develop dementia earlier in the disease course than PD_*GBA_wild type*_ prompted the community to perform a large multicenter analysis across 11 centers evaluating *GBA* mutations in 721 cases with DLB, which represents a clinico-histopathological continuum to PD. With an even higher OR of 8.28, *GBA* mutations are also strongly associated with DLB. Similar to PD, *GBA* mutations also predispose to an earlier age at onset and more pronounced disease severity/progression in DLB (Nalls et al., 2013). This study further supported *GBA* mutations as a significant genetic risk factor for synucleinopathies and confirmed the overall impression that *GBA*-related Parkinsonism predisposes to an increased incidence of dementia.

### *GBA* Mutations and Multisystem Atrophy

The higher prevalence of autonomic dysfunction and the link to alpha-synuclein pathology as discussed further on raised the idea that *GBA* variants might also be associated with increased risk to develop MSA. However, studies in MSA revealed conflicting results, both in clinically diagnosed as well as in autopsy-confirmed cases. One large study investigated 969 MSA patients with Japanese, European, and North American background and found an overall OR of 2.44. Notably, the authors reported a significant association between *GBA* variants and MSA-C phenotype (Mitsui et al., 2015). Another study with autopsy-confirmed MSA and Alzheimer patients reported a higher frequency of *GBA* variants in the MSA group compared to the AD group (Sklerov et al., 2017). However, a larger study with autopsy-proven MSA cases could not show an association of *GBA* variants with MSA (Segarane et al., 2009). Moreover, several studies with clinical diagnosis of MSA also found no clear association (Srulijes et al., 2013; Sun et al., 2013; Asselta et al., 2014).

### *GBA*-Associated Pathomechanism and Histopathology

**FIGURE 1:**
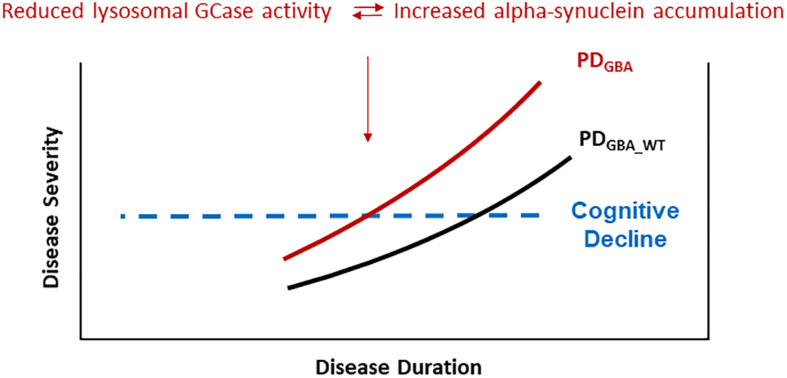
PD patients carrying pathogenic *GBA* mutations show a more rapid progression of motor symptoms, specifically of cognitive decline. These clinical characteristics seem to be driven by accelerated alpha-synuclein aggregation and propagation due to a self-inforcing bidirectional pathogenic loop between GCase deficiency and alpha-synuclein accumulation. Especially the high risk of cognitive decline in yet cognitively intact PD_*GBA*_ patients provides a defined window of opportunity for modifying treatment options targeting alpha-synuclein.

Evidence from cell models favors the hypothesis that *GBA* mutations result in disrupted trafficking of GCase from the ER to Golgi and in lower lysosomal GCase enzyme activity which in turn cause a build-up of lysosomal glucosylceramides and impair alpha-synuclein degradation (Mazzulli et al., 2011). Highlighting the role of lysosomal dysfunction in the pathogenesis of PD, results from postmortem brain tissue and IPS cell-derived neurons show that reduced lysosomal GCase activity is paralleled by increased levels of alpha-synuclein, not only in PD_*GBA*_ but, to a lesser degree, also in cases with PD_*GBA_wild type*_ (Gegg et al., 2012; Murphy et al., 2014; Schondorf et al., 2014; Moors et al., 2019). Moreover, recent data in human midbrain dopaminergic neurons suggest that conformational changes of alpha-synuclein toward an aggregation-prone pattern can be even induced by the presence of glycosphingolipids irrespective of an underlying mutation in the *GBA* gene (Zunke et al., 2018). More specifically, it was suggested that lysosomal GCase and alpha-synuclein are linked in a bidirectional pathogenic loop in synucleinopathies as shown in cell cultures and in induced-pluripotent stem (IPS) cell-derived dopaminergic midbrain neurons: (1) Functional loss of GCase activity compromises lysosomal degradation of alpha-synuclein and causes its aggregation due to reduced lysosomal chaperone-mediated autophagy. (2) Alpha-synuclein itself inhibits the activity of GCase (Mazzulli et al., 2011; Schondorf et al., 2014). Consequently, PD_*GBA*_ fulfill both conditions of this bidirectional loop in parallel leading to a self-reinforcing mechanism. Thereby, alpha-synuclein aggregation and propagation might be enhanced which possibly explains the wide-spread neocortical Lewy body pathology observed in postmortem brain tissue of PD_*GBA*_ (Neumann et al., 2009; Gundner et al., 2019; Moors et al., 2019). These pathomechanistic aspects in turn offer a reasonable explanation for the more severe and more rapid disease progression seen in the prodromal and in the manifest phase in PD_*GBA*_ (Figure 1).

### *GBA*-Associated Biomarker Profiles in Patient-Derived Biofluids

Despite this clear experimental evidence, we often fail to translate these findings into clinical research with patient cohorts, and we lack to confirm the impact of genetic mutations on biochemical profiles in patient-derived biomaterial. Moreover, we need to evaluate whether such profiles might be suitable as biochemical readout for target engagement.

Heterozygous variants in the *GBA* gene are associated with lower levels of lysosomal GCase enzyme activity using a variety of assays in different patient-derived biofluids including blood and CSF (Schondorf et al., 2014; Alcalay et al., 2015; Paciotti et al., 2019). Similar to the findings in brain tissue, GCase activity is also reduced in PD patients without *GBA* variant, albeit to a lesser degree (Parnetti et al., 2017).

As we have no reliable imaging marker available to assess the cerebral load of alpha-synuclein *in vivo*, research has focused on CSF. Importantly, it is widely discussed whether CSF profiles of alpha-synuclein species reflect alpha-synuclein pathology in the brain. Analyses in sporadic PD demonstrated CSF levels of total alpha-synuclein to be decreased in PD compared to healthy controls (Malek et al., 2014; Mollenhauer et al., 2019). However, CSF levels of total alpha-synuclein do not correlate with motor-associated disease progression. These findings imply that CSF levels of total alpha-synuclein are not suitable to monitor motor progression in the clinically manifested disease phase and/or that the nature of progression is too slow. Since prodromal PD subjects with hyposmia and/or REM-sleep behavior disorder already show decreased CSF levels of alpha-synuclein (Mollenhauer et al., 2019), one might argue that this phenomenon develops early in the disease and does not parallel with the manifest disease phase.

Given the specific mechanistic link between *GBA* and alpha-synuclein, it is tempting to speculate whether PD_*GBA*_ represent a proxy for alpha-synuclein-driven CSF profiles. Indeed, we could show that PD_*GBA*_ present with lower CSF levels of total alpha-synuclein compared to healthy controls and also compared to PD_*GBA_wild type*_. Importantly, PD_*GBA*_ with severe variants show the lowest mean values (Lerche et al., 2020).

As PD_*GBA*_ present accelerated cognitive decline, this subgroup of PD patients represent a good model to study biochemical profiles in CSF that might be associated with cognitive impairment. In general, limbic and/or cortical Lewy body pathology is hypothesized to be the main substrate driving cognitive decline in sporadic PD (Aarsland et al., 2005). In more recent years, it became clear that a considerable proportion of sporadic PD patients who developed dementia in their disease course show concomitant amyloid-beta and tau pathology at autopsy in addition to the typical Lewy-body pathology (Halliday et al., 2008; Compta et al., 2011). Correspondingly, reduced CSF levels of amyloid-beta_1_____42_ and/or elevated CSF levels of total-tau and phospho-tau have been reported to be associated with cognitive impairment in sporadic PD (Brockmann et al., 2015a, 2017; Kang et al., 2016; Lerche et al., 2019b). However, this seems not to be the case in PD_*GBA*_ as CSF levels of Aβ_1__–__42_, t-TAU, and p-Tau are similar to those seen in healthy control individuals, whereas levels of alpha-synuclein were lower. These findings suggest that the prominent cognitive impairment in PD_*GBA*_ is not associated with amyloid-beta or tau pathology but might be driven by alpha-synuclein aggregation.

Based on the genetic link between *GBA* mutations and DLB, it was tempting to speculate whether findings of CSF alpha-synuclein profiles in PD_*GBA*_ patients can be also detected in DLB patients carrying a *GBA* mutation. Similar to PD, *GBA* mutations are associated with decreased CSF levels of total alpha-synuclein in DLB patients. Again, these findings seem dependent on *GBA* mutation severity and were most pronounced in DLB_*GBA*_ patients with severe mutations (Lerche et al., 2019a).

These *in vivo* data seem to confirm findings from cell models and postmortem analysis: lower GCase activity is associated with prominent CSF profiles of total alpha-synuclein representing a mirror of greater cerebral Lewy pathology in PD_*GBA*_ and DLB_*GBA*_ patients (Neumann et al., 2009; Gundner et al., 2018; Moors et al., 2018). Yet, a substantial inter-individual variability and overlap with healthy controls is seen so that CSF levels of total alpha-synuclein are not ideal to be used as a single biomarker. More recently, real−time quaking−induced conversion (RT−QuIC) and protein misfolding cyclic amplification (PMCA) have been successfully implemented to evaluate alpha-synuclein seeding capacities. These assays are based on the conversion of monomeric substrate protein into β−sheet−rich aggregates by seeding with small amounts of protein aggregates (Fairfoul et al., 2016; Shahnawaz et al., 2017). As this method is highly sensitive and specific for alpha-synuclein aggregation, PD and DLB patients with *GBA* mutations would be prime candidates to be assessed with this assay.

## Discussion and Outlook

PD patients carrying pathogenic *GBA* mutations show a faster motor progression and cognitive decline. Importantly, the higher risk of cognitive decline is not associated with amyloid-β pathology (e.g., CSF Abeta_1_____42_ levels) as shown instead in sporadic PD without *GBA* mutations. Postmortem studies show that lower levels of lysosomal GCase activity are associated with greater alpha-synuclein pathology in PD, PDD, and PD_*GBA*_ brains. This has been confirmed by *in vivo* studies showing that PD and DLB patients carrying pathogenic *GBA* mutations had reduced levels of lysosomal GCase activity paralleled by lower CSF levels of total alpha-synuclein (possibly mirroring greater Lewy pathology in the brain). Based on these results, high amounts of aggregated alpha-synuclein could play a pivotal role in cognitive decline in PD_*GBA*_ (Figure 1).

So far, drugs aiming at slowing disease progression in neurodegenerative diseases failed. One reason that could be discussed is that they were administered in manifest disease stages where brain pathology is too advanced. PD_*GBA*_ patients who are cognitively intact represent a high-risk population to develop PD-associated dementia and thus provide a defined window of opportunity for treatment aiming to delay cognitive decline. Thereby, starting a treatment able to reduce the propagation of aggregated alpha-synuclein in this population would overcome the challenge of starting intervention early enough. As alpha-synuclein aggregation seems to be the main driver of dementia in PD_*GBA*_, this specific population represents a role model to study the effect of alpha-synuclein lowering treatment strategies such as monoclonal antibodies targeting aggregated alpha-synuclein.

A more complex picture is now beginning to emerge and points toward a central role for GCase activity not only in *GBA*-associated PD but also in sporadic as well as other genetic forms. Postmortem brain tissue analyses, patient-derived IPS-cell models, and CSF studies show decreased levels of GCase activity paralleled by alpha-synuclein accumulation in wild-type PD patients and in PD patients with mutations in *LRRK2*, *parkin*, and *DJ-1.* Interestingly, LRRK2 kinase activity seems to regulate GCase activity in an inverse pattern (Parnetti et al., 2017; Burbulla et al., 2019; Ysselstein et al., 2019). These recent findings highlight not only the importance of lysosomal dysfunction in the pathophysiology of PD_*GBA*_ but the significance of this pathway for PD in general. Thereby, one could imagine that lysosomal-targeted treatment options developed for PD_*GBA*_ (Mullin et al., 2020) might be also beneficial for other PD subgroups in which lysosomal dysfunction driving alpha-synuclein accumulation plays a major role. However, at this point we lack direct comparisons with the same assay/methodology evaluating the degree of GCase activity reduction, lysosomal dysfunction, and consecutive alpha-synuclein accumulation between wild-type and different genetic forms of PD (*GBA, LRKK2, Parkin, PINK*, etc.).

## Author Contributions

The author confirms being the sole contributor of this work and has approved it for publication.

## Conflict of Interest

The author declares that the research was conducted in the absence of any commercial or financial relationships that could be construed as a potential conflict of interest.
